# Supplementary material for: Early planetesimal differentiation and late accretion shaped Earth’s nitrogen budget
Source: Nat Commun. 2024 May 16;15:4169. doi: 10.1038/s41467-024-48500-0 (PMC11099130; doi:10.1038/s41467-024-48500-0)
Supplement: Supplementary file 1 — Supplementary Information [file 41467_2024_48500_MOESM1_ESM.pdf]

Supplementary materials for " Early planetesimal differentiation and late accretion shaped Earth's nitrogen budget"

Wenzhong Wang[1,2,3,4*], Michael J. Walter[3], John P. Brodholt[4,5], Shichun Huang[6]

[1]Deep Space Exploration Lab/School of Earth and Space Sciences, University of Science and Technology of China, Hefei, Anhui 230026, China

[2]CAS Center for Excellence in Comparative Planetology, University of Science and Technology of China, Anhui, China.

[3]Earth and Planets Laboratory, Carnegie Institution for Science, Washington, DC 20015, USA

[4]Department of Earth Sciences, University College London, London WC1E 6BT, United Kingdom

[5]The Centre of Planetary Habitability, University of Oslo, Oslo, Norway

[6]Department of Earth & Planetary Sciences, University of Tennessee at Knoxville, Knoxville, United States

*Corresponding author. Email: wwz@ustc.edu.cn

Including:

Supplementary Notes

Supplementary Table 1

Supplementary Figures 1 to 16

Nitrogen isotope compositions of chondrites

The $\delta^{15}N$ of enstatite chondrites were investigated by two previous studies, Kung and Clayton (1978)[1] and Grady et al. (1986)[2]. Kung and Clayton (1978)[1] measured the whole-rock $\delta^{15}N$ of five enstatite chondrites (Abee, Indarch, Hvittis, Pillistfer, and Daniel's Kuil), which ranges from -24 to -43 ‰. Grady et al. (1986)[2] determined the whole-rock $\delta^{15}N$ of ten enstatite chondrites (Abee, Indarch, Kota-Kota, South Oman, St. Mark's, Atlanta, Daniel's Kuil, Khairpur, N.W. Forrest, Yilmia) using stepped pyrolysis and combustion extractions. The results could be significantly affected by the sample weight and the analytical method. For instance, the stepped combustion measurements show that the $\delta^{15}N$ of the South Oman meteorite is -27.4 ‰ when a 0.406 mg sample was used, while it is -16.6 ‰ when a 4.545 mg sample was used. For the St. Mark's meteorite, the stepped combustion analysis gives a $\delta^{15}N$ of -6.3 ‰, while its $\delta^{15}N$ is -12.2 ‰ using the stepped pyrolysis extraction. Regardless of the sample weight, the stepped combustion measurements show that the $\delta^{15}N$ values range from -30 to -6 ‰, while the stepped pyrolysis measurements give a range of $\delta^{15}N$ from -47 to +0.6 ‰.

When all measurements are put together[1,2], the $\delta^{15}N$ of enstatite chondrites ranges from -47 to -6 ‰, except for one stepped pyrolysis measurement for South Oman (+0.6 ‰). Twenty-eight out of the thirty measurements are within the range of -47 to -10 ‰. The South Oman meteorite also has an unusual whole-rock $\delta^{13}C$ value (over 15 ‰ lighter than most other enstatite chondrites)[2], probably due to the presence of terrestrial contaminants. If the data measured using the largest sample weight is used, the $\delta^{15}N$ ranges from -47 to -10 ‰. If the average value of all the single measurements is used for each meteorite sample, the $\delta^{15}N$ of these ten enstatite chondrites ranges from -36 to -15 ‰.

The $\delta^{15}N$ of carbonaceous chondrites show large differences between different groups[3,4]. The CI chondrites have a whole-rock $\delta^{15}N$ range of +31 to +52 ‰, and the CM carbonaceous chondrites have $\delta^{15}N$ of -16 to +56 ‰, most of which are positive[3,4]. In contrast, most CV carbonaceous chondrites have negative $\delta^{15}N$ values[3,4], though the $\delta^{15}N$ ranges from -45 to +24 ‰. The $\delta^{15}N$ of CO carbonaceous chondrites range from -30 to +13 ‰ (refs. [3,4]). When all data of carbonaceous chondrites are put together, ~ 75% of carbonaceous chondrites have $\delta^{15}N$ of +10 to +56 ‰.

The $\delta^{15}N$ of ordinary chondrites were measured by Kung and Clayton (1978)[1] and Hashizume and Sugiura (1995)[5]. The $\delta^{15}N$ of six ordinary chondrites in Kung and Clayton (1978)[1] range from -3 to +20 ‰, and the $\delta^{15}N$ of twenty-one ordinary chondrites in Hashizume and Sugiura (1995)[5] range from -2 to +26 ‰. Among all measured data, only two values are negative and most of them are higher than +10 ‰.

Supplementary Table 1. Cell parameters, volumes of simulated boxes for silicate and metallic melts and average force constants of N atoms in these melts at different pressures. The uncertainties of force constants refer to the standard error.

Melt	Cubic cell size (Å)	Volume (Å^3)	Pressure (GPa)	Average force constant of N atom <*F*> (N/m)	Uncertainty (N/m)
$Mg_{32}Si_{32}O_{96}N_2$	12.6	2000.376	5.6	752.5	13.1
	11.6	1560.896	23.7	774.9	7.7
	11.05	1349.233	45.0	756.5	7.0
	10.6	1191.016	77.7	773.1	9.6
	10.4	1124.864	99.6	770.0	9.5
$Mg_{30}NaCa_2Fe_4Si_{24}Al_3O_{89}N_2$	12.5	1953.125	5.3	698.7	9.8
$Mg_{32}Si_{32}O_{96}NH_3$	12.6	2000.376	5.5	366.9	6.4
	11.6	1560.896	23.4	437.6	7.3
	11.05	1349.233	43.6	476.6	6.5
	10.6	1191.016	76.5	529.9	7.6
	10.4	1124.864	97.7	576.1	7.3
$Mg_{30}NaCa_2Fe_4Si_{24}Al_3O_{89}NH_3$	11.5	1520.875	23.3	397.0	5.8
$Mg_{32}Si_{32}O_{96}N_3H_9$	12.65	2024.285	6.2	416.1	4.4
$Fe_{98}N_2$	10.66	1211.355	0.7	168.8	3.2
	10.25	1076.890	19.5	191.3	3.8
	10.0	1000.000	39.2	216.9	4.3
	9.8	941.192	60.6	232.9	4.6
	9.55	870.984	98.1	287.4	3.3
$Fe_{87}Ni_4Si_6S_2C_2OH_5N_2$	10.35	1108.719	25.1	204.6	3.4
N_2 molecule	20			805.6	-

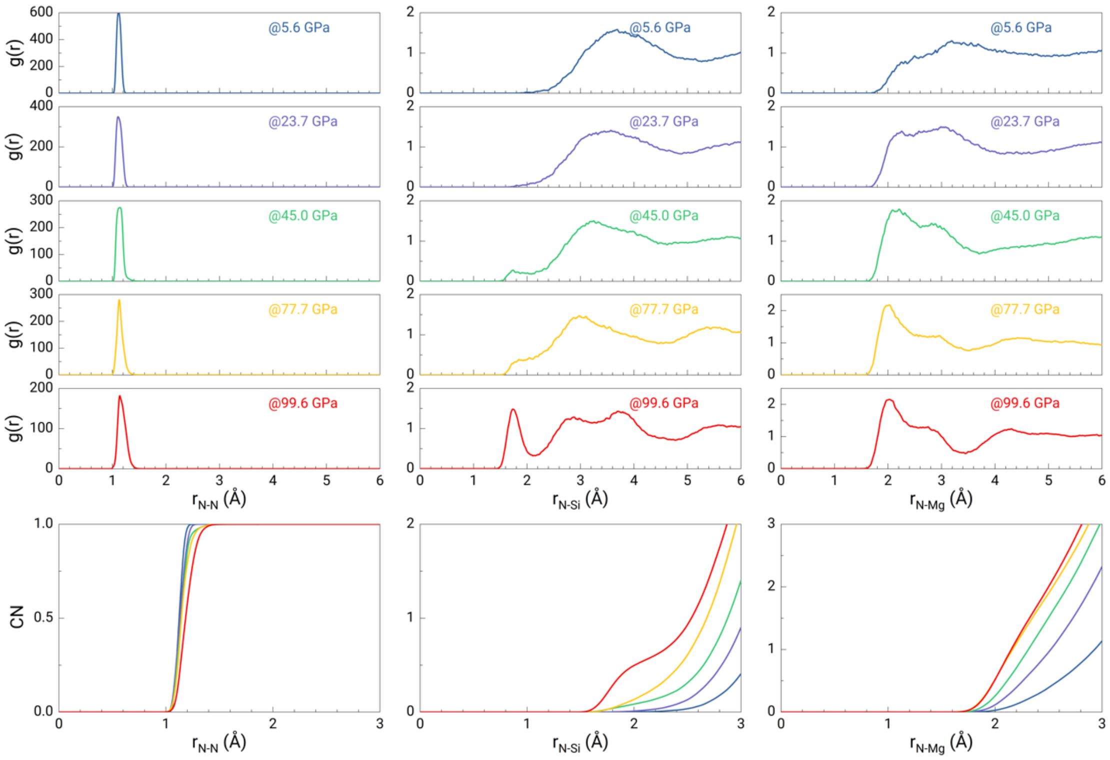

Supplementary Figure 1. Radial distribution functions g(r) and coordination numbers (CN) for the N-N, N-Si, and N-Mg pairs in the $Mg_{32}Si_{32}O_{96}N_2$ silicate melt at different pressures.

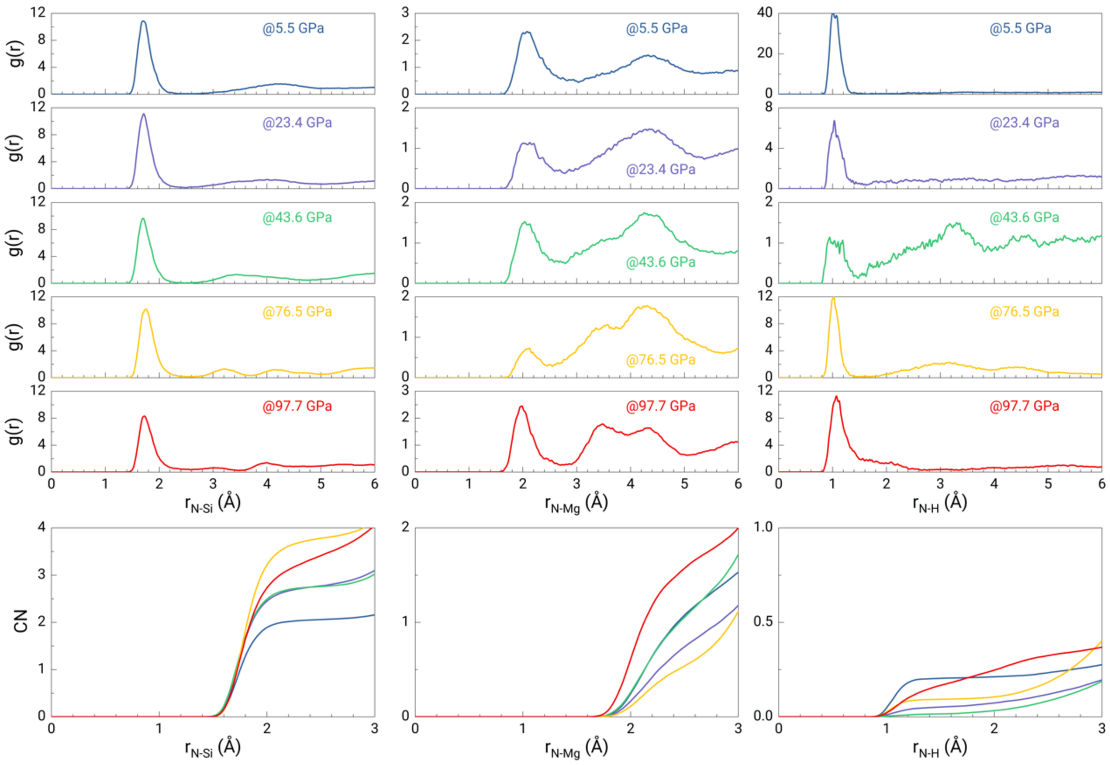

Supplementary Figure 2. Radial distribution functions g(r) and coordination numbers (CN) for the N-Si, N-Mg, and N-H pairs in the $Mg_{32}Si_{32}O_{96}NH_3$ silicate melt at different pressures.

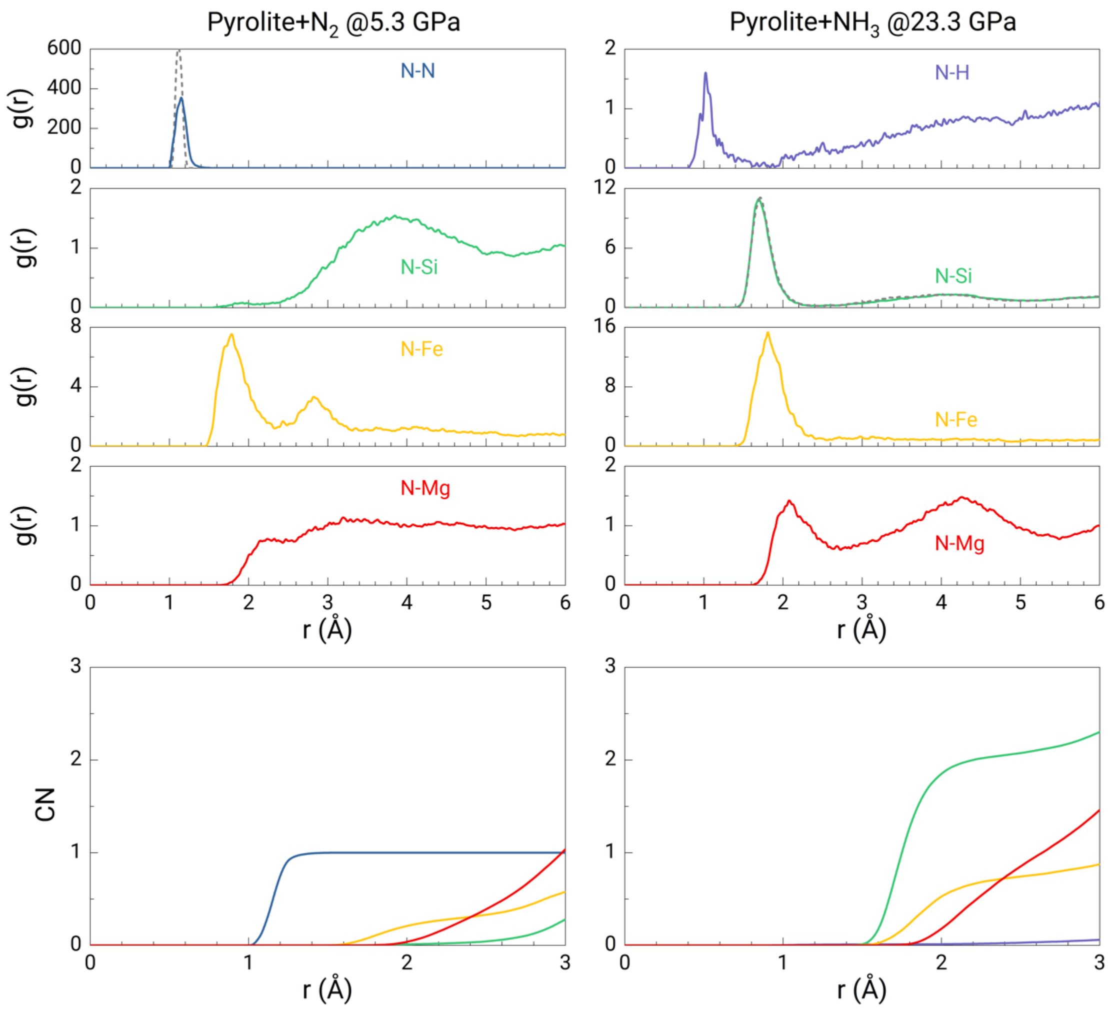

Supplementary Figure 3. Radial distribution functions g(r) and coordination numbers (CN) for the N-N/H, N-Si, N-Fe, and N-Mg pairs in the $Mg_{30}NaCa_2Fe_4Si_{24}Al_3O_{89}N_2$ (pyrolite+N_2) and $Mg_{30}NaCa_2Fe_4Si_{24}Al_3O_{89}NH_3$ (pyrolite+NH_3) silicate melts. The dash lines in the left and right figures represent the N-N distance in the $Mg_{32}Si_{32}O_{96}N_2$ melt and the N-Si distance in the $Mg_{32}Si_{32}O_{96}NH_3$ melt, respectively.

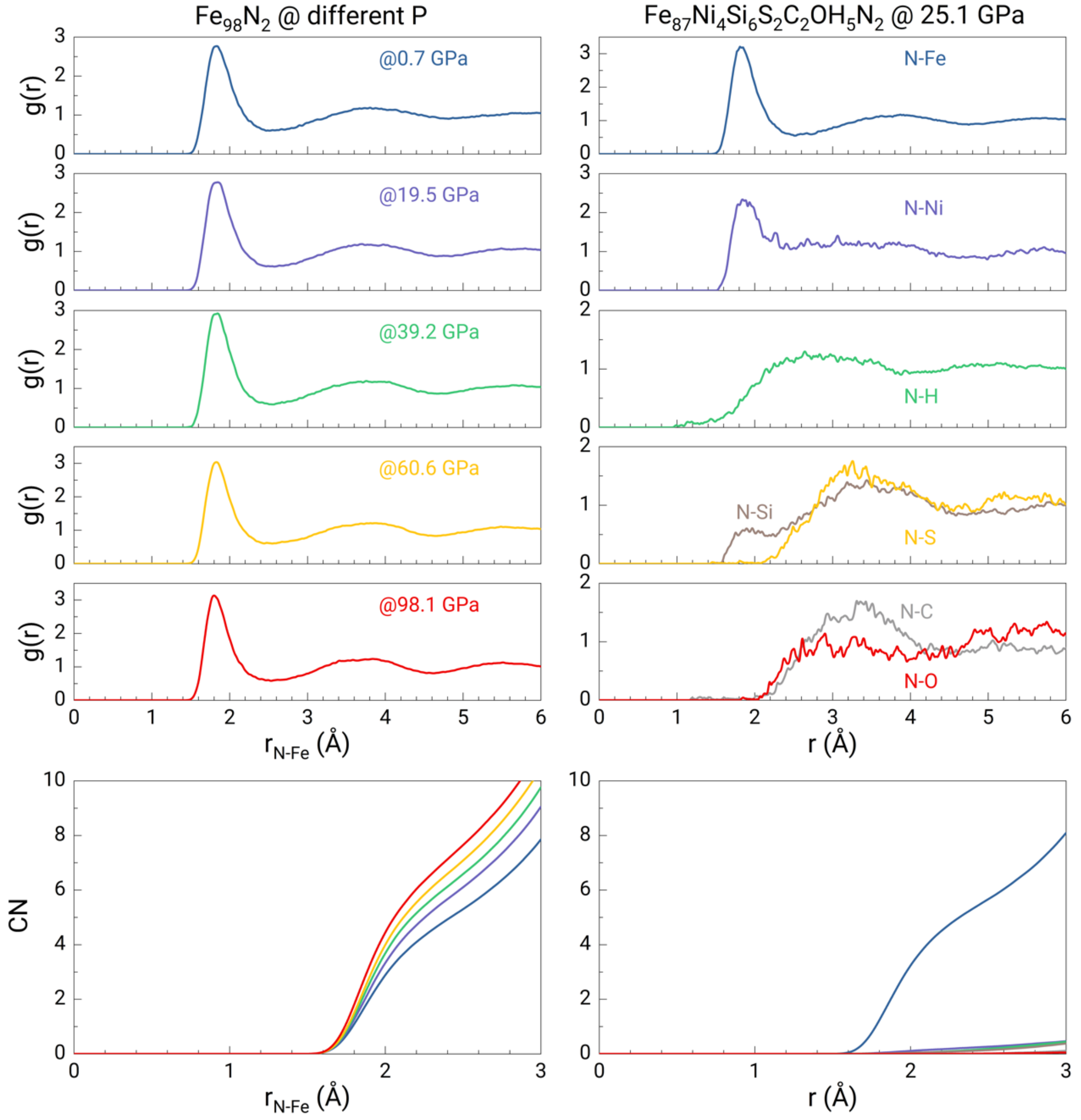

Supplementary Figure 4. Radial distribution functions g(r) and coordination numbers (CN) for the N-Fe in the $Fe_{98}N_2$ metallic melt at different pressures and for the N-Fe, N-Ni, N-H, N-Si, N-S, N-C, and N-O pairs in the $Fe_{87}Ni_4Si_6S_2C_2OH_5N_2$ melt.

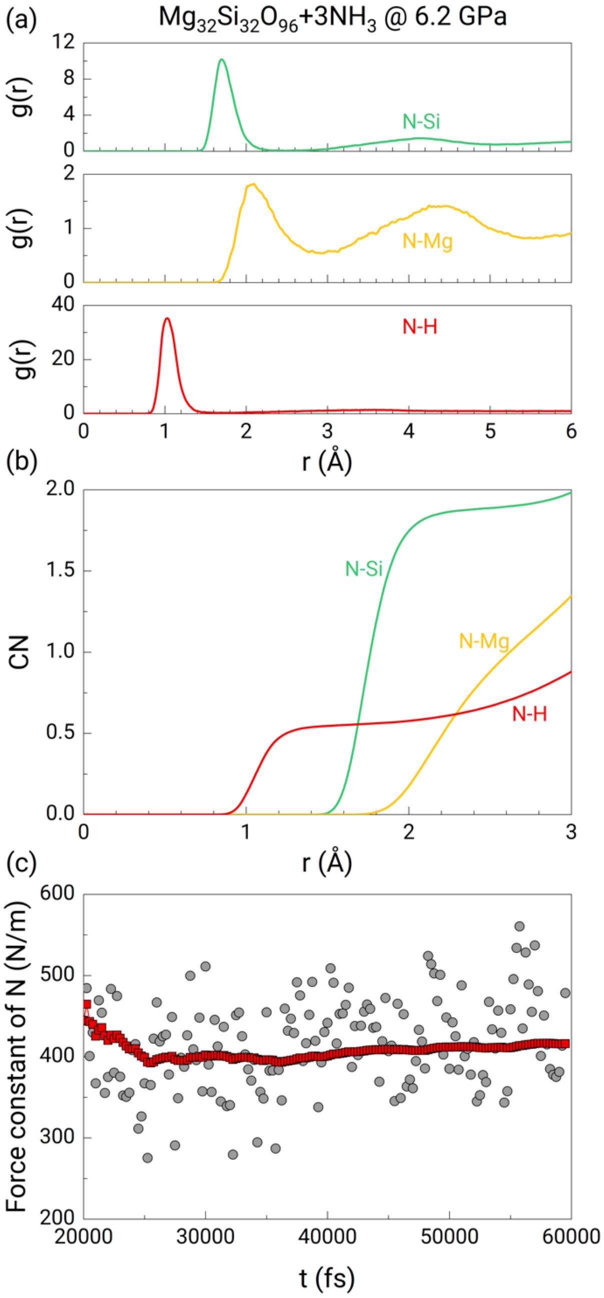

Supplementary Figure 5. Structural properties and force constant of N in the $Mg_{32}Si_{32}O_{96}N_3H_9$ silicate melt. (a) radial distribution functions g(r) and (b) coordination numbers (CN) for the N-Si, N-Mg, and N-H pairs. (c) the force constants of N atom in captured snapshots (grey circles) and their cumulative averages (red squares) in the time domain.

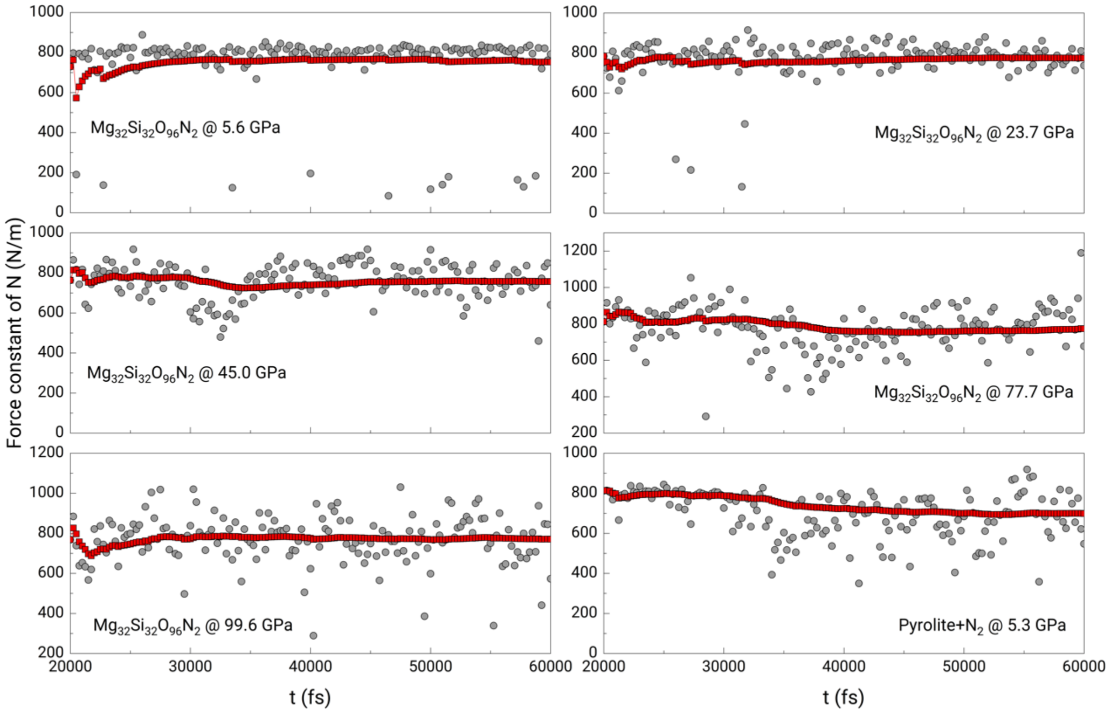

Supplementary Figure 6. The force constants of N atom in captured snapshots (grey circles) from $Mg_{32}Si_{32}O_{96}N_2$ and $Mg_{30}NaCa_2Fe_4Si_{24}Al_3O_{89}N_2$ (pyrolite+N_2) silicate melts and their cumulative averages (red squares) in the time domain.

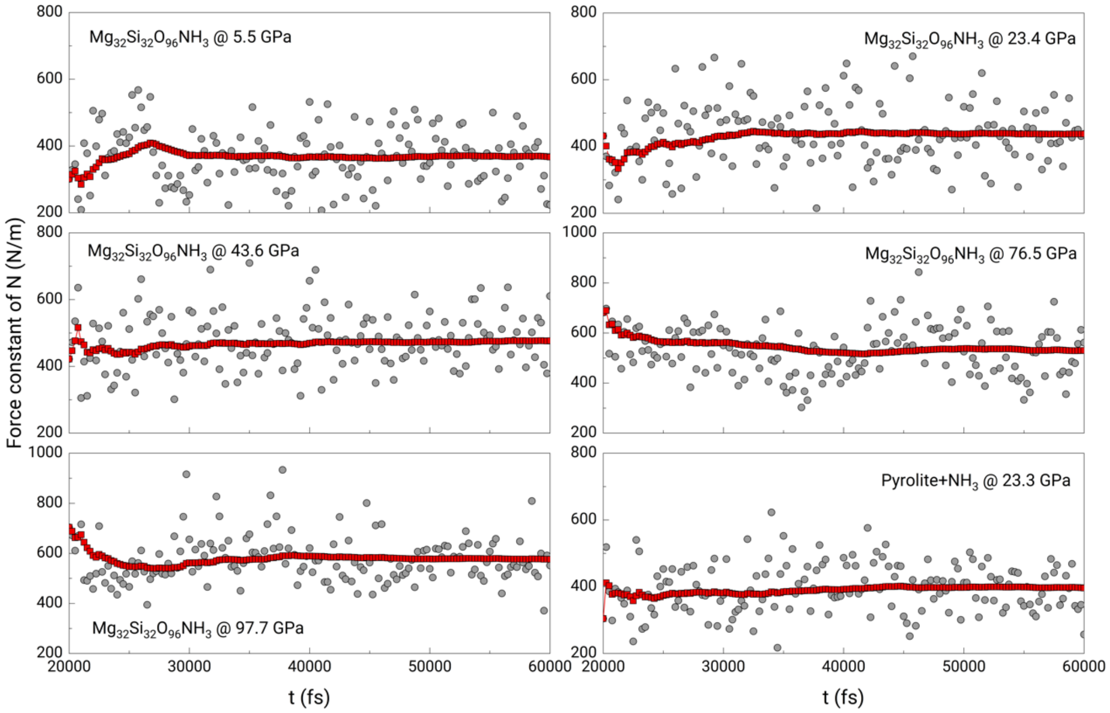

Supplementary Figure 7. The force constants of N atom in captured snapshots (grey circles) from $Mg_{32}Si_{32}O_{96}NH_3$ and $Mg_{30}NaCa_2Fe_4Si_{24}Al_3O_{89}NH_3$ (pyrolite+NH_3) silicate melts and their cumulative averages (red squares) in the time domain.

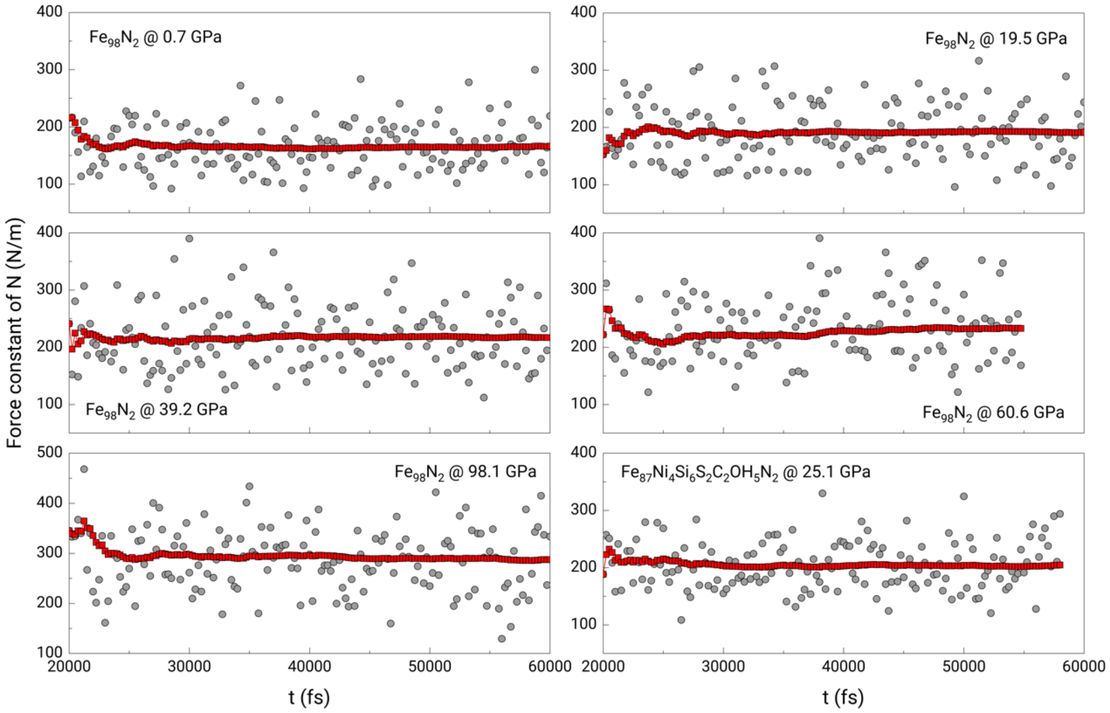

Supplementary Figure 8. The force constants of N atom in captured snapshots (grey circles) from $Fe_{98}N_2$ and $Fe_{87}Ni_4Si_6S_2C_2OH_5N_2$ metallic melts and their cumulative averages (red squares) in the time domain.

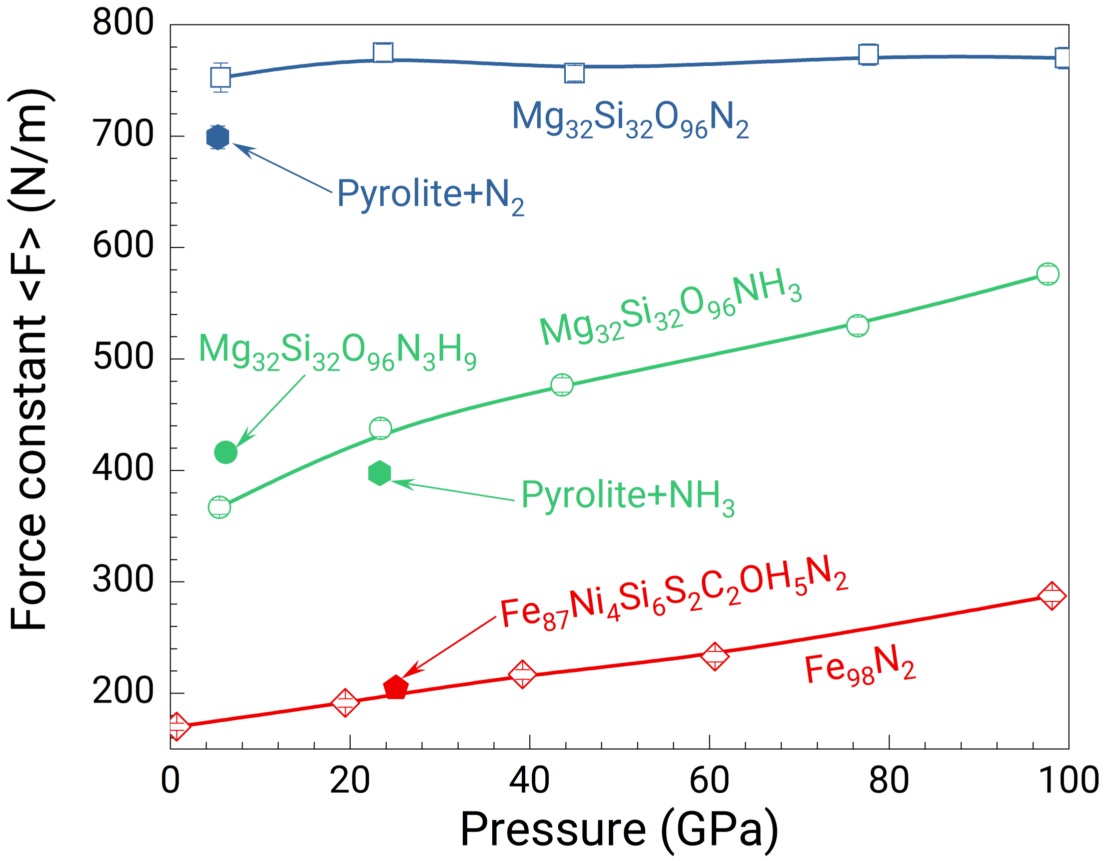

Supplementary Figure 9. Average force constants <*F*> of N in silicate and metallic melts at different pressures. $Mg_{32}Si_{32}O_{96}N_2$ and $Mg_{32}Si_{32}O_{96}NH_3$ represent the N-bearing silicate melts under relatively oxidizing and reducing conditions, respectively.

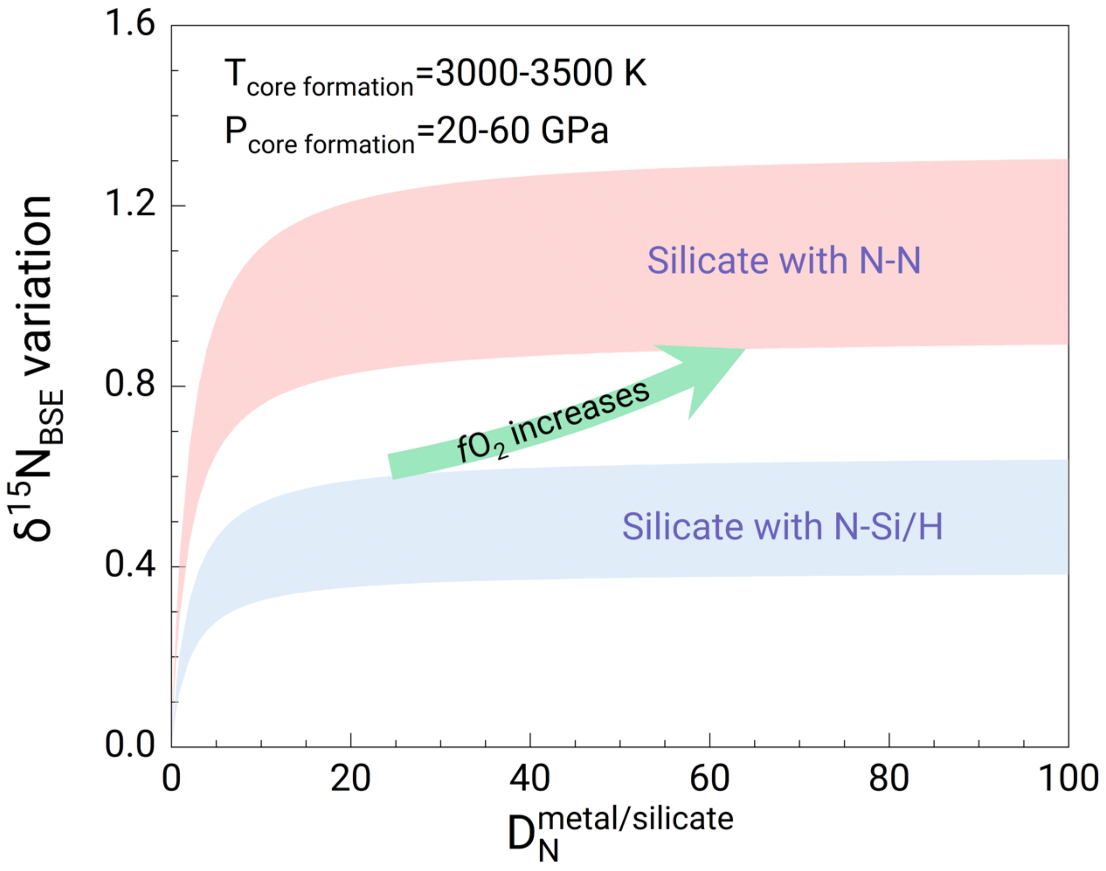

Supplementary Figure 10. The variation of $\delta^{15}N$ in bulk silicate reservoir (mantle + crust + atmosphere) caused by core-mantle differentiation using an equilibrium fractionation model. The equilibrium N isotope fractionation between silicate and metallic melts ($10^3\ln\alpha_{silicate\text{-}metal}$) as a function of temperature and pressure under relatively oxidized and reduced conditions are shown in Fig. 1.

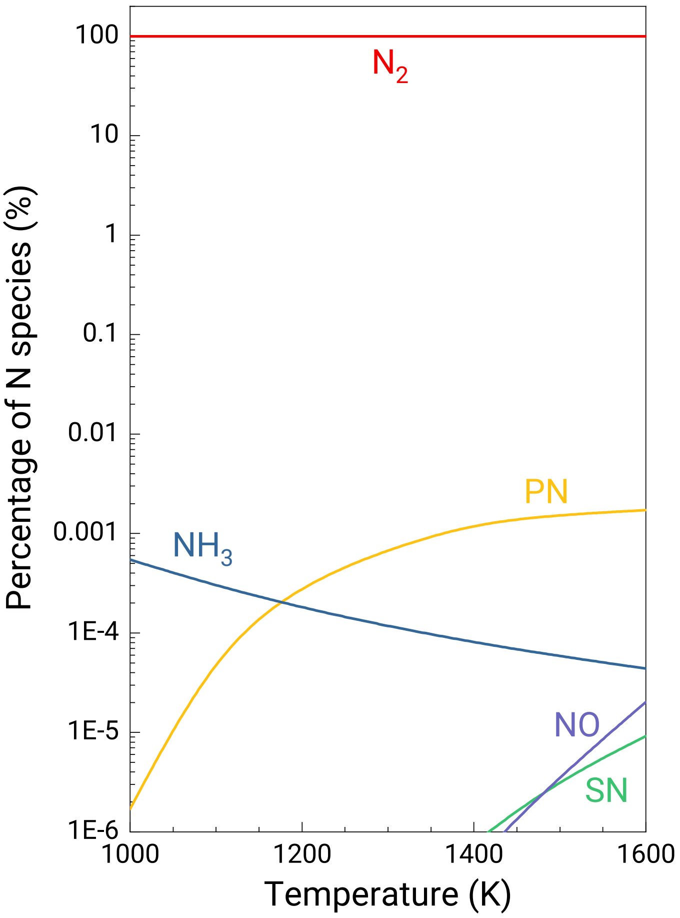

Supplementary Figure 11. the molar percentages of N species in the vapor phase during planetesimal evaporation in the presence of nebular H_2. Thermodynamic calculations were conducted using the GRAINS code [6] with solar abundances for the elements [7]. The results show that regardless of the H concentration in the system, N in the vapor phase dominantly occurs as N_2 with a percentage of > 99.9 %

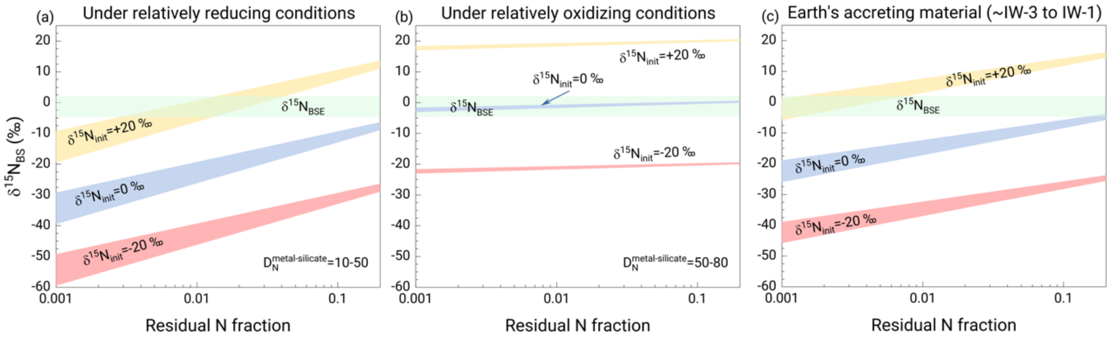

Supplementary Figure 12. Effects of planetesimal evaporation and core formation on the $\delta^{15}N$ of rocky planets. An equilibrium N isotope fractionation model was adopted to estimate the effect of core formation. The figure legends are the same as those of Fig. 2.

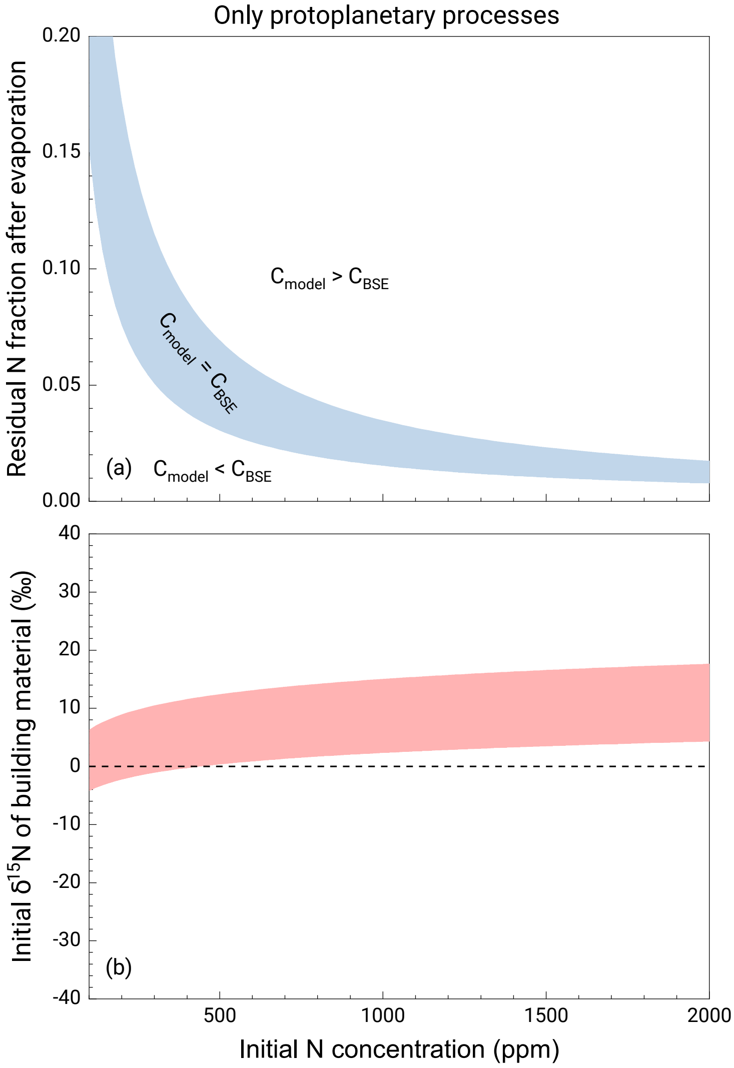

Supplementary Figure 13. Ranges of the initial N concentration and $\delta^{15}N$ of the building materials for Earth that can reproduce the $\delta^{15}N_{BSE}$ when only planetary processes are considered. (a) the residual N fraction after evaporation versus the initial N concentration (C_{init}). The blue region represents the range that can reproduce the C_{BSE}. (b) the required initial $\delta^{15}N$ of the building materials for Earth. The initial $\delta^{15}N$ should be positive up to +14 ‰ if C_{init} is 500-1500 ppm.

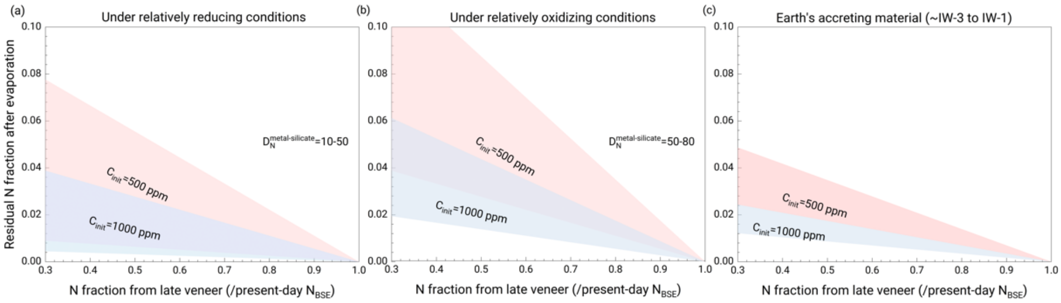

Supplementary Figure 14. Reproducing the N concentration in the BSE using planetary processes (evaporation + core formation) and a late veneer. The residual N fraction after evaporation versus the N fraction from a late veneer under (a) relatively reducing conditions ($D_N^{metal\text{-}silicate}$=10-50); (b) relatively oxidizing conditions ($D_N^{metal\text{-}silicate}$=50-80); and (c) at the oxygen fugacity (log fO_2) of ~IW-3 to IW-1 for Earth's accreting materials. The red and blue regions represent the ranges than can reproduce the C_{BSE} when the initial N concentration (C_{init}) is 500 and 1000 ppm, respectively. The less N remains after evaporation, the more N should be added by a late veneer.

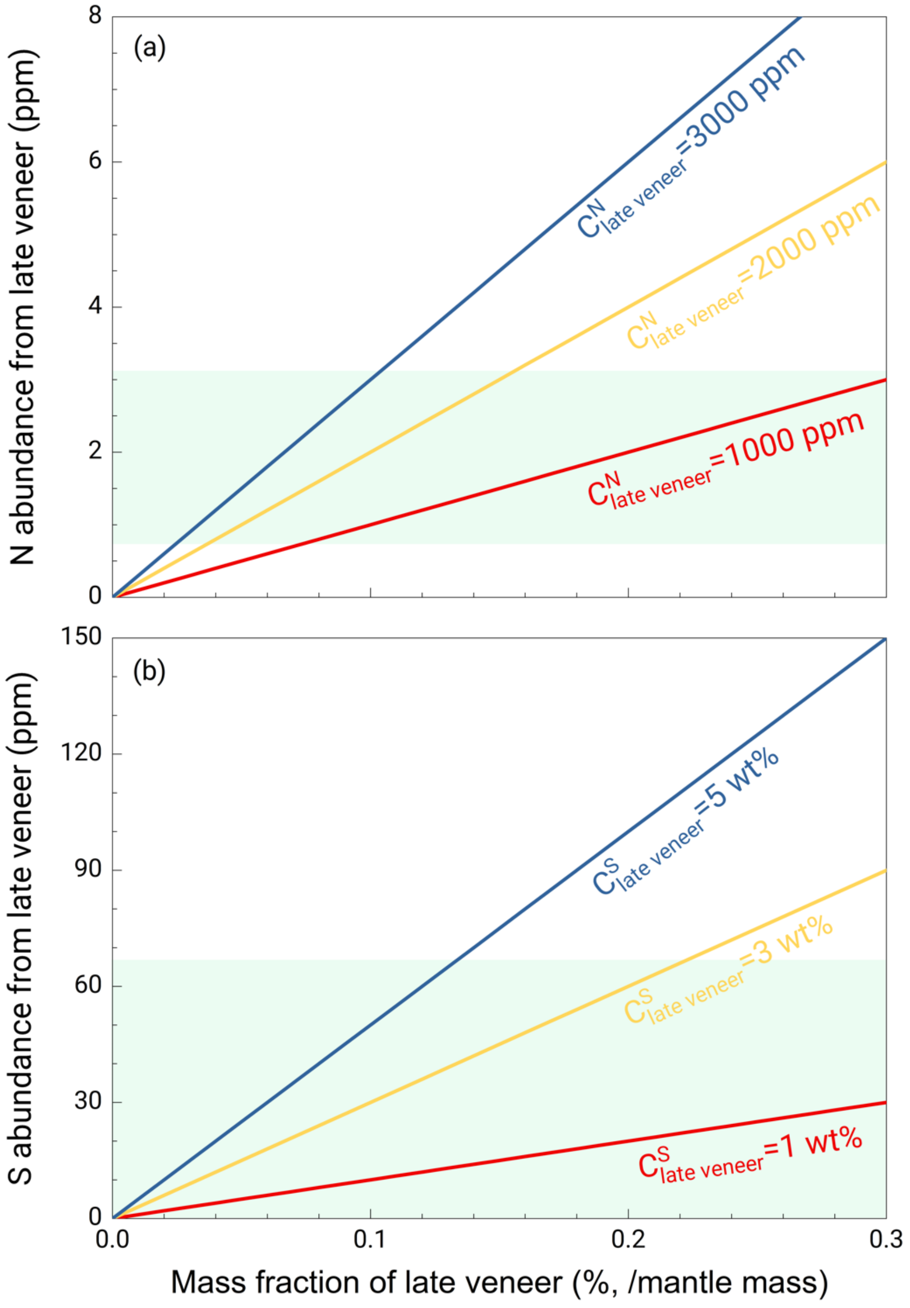

Supplementary Figure 15. Nitrogen and sulfur abundances in the BSE contributed by late veneer versus the mass fraction of late veneer. The N isotopic evidence shows that 30-100% of N in the modern BSE should be added by a carbonaceous-chondrite-like late veneer (the green region in (a)), while the S isotopic evidence suggests that the amount of S added by a late veneer should not exceed ~30% S of the modern BSE (the green region in (b)). Both scenarios demonstrate that the fraction of late veneer would amount to a maximum estimate of 0.2% of the mass of Earth's mantle.

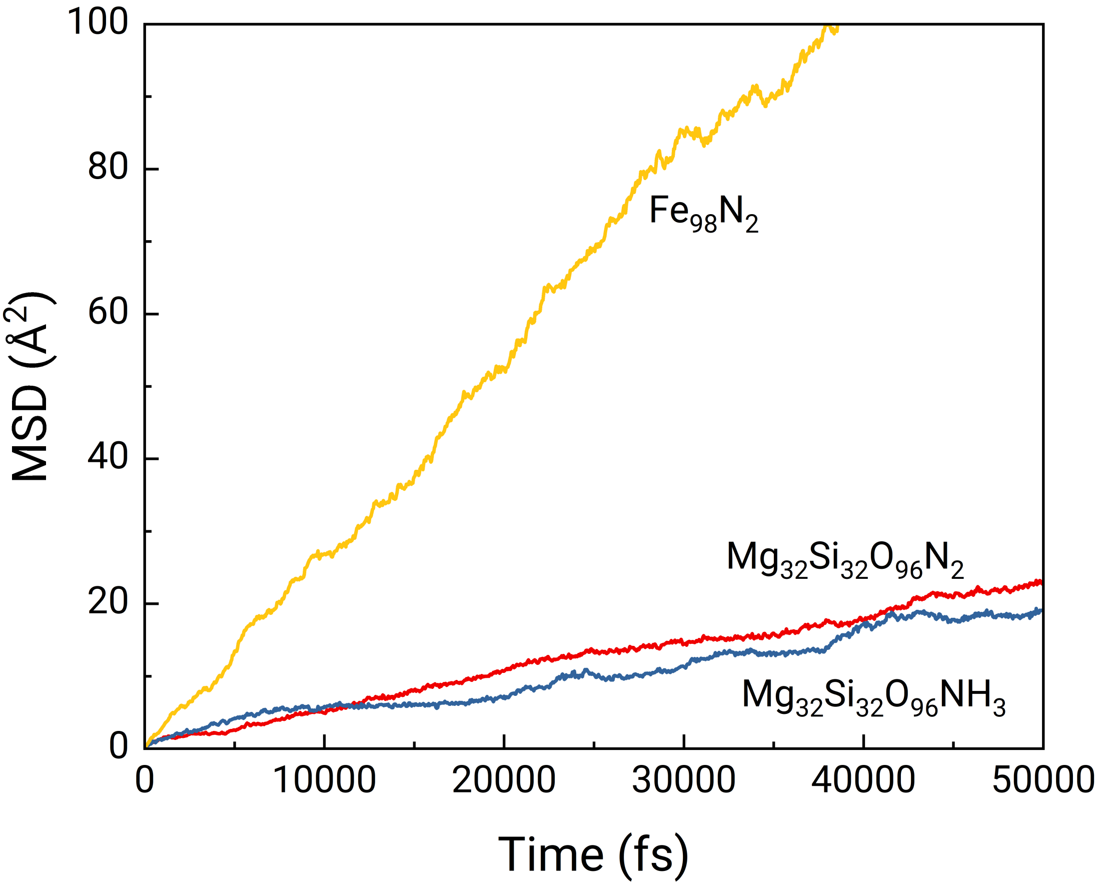

Supplementary Figure 16. Mean square displacements (MSD) of $Mg_{32}Si_{32}O_{96}N_2$, $Mg_{32}Si_{32}O_{96}N_2$, and $Fe_{98}N_2$ systems.

Refences

1. Kung, C.-C. & Clayton, R. N. Nitrogen abundances and isotopic compositions in stony meteorites. *Earth Planet. Sci. Lett.* **38**, 421–435 (1978).
2. Grady, M. M., Wright, I. P., Carr, L. P. & Pillinger, C. T. Compositional differences in enstatite chondrites based on carbon and nitrogen stable isotope measurements. *Geochim. Cosmochim. Acta* **50**, 2799–2813 (1986).
3. Alexander, C. M. O. *et al.* The Provenances of Asteroids, and Their Contributions to the Volatile Inventories of the Terrestrial Planets. *Science (80-.).* **337**, 721–723 (2012).
4. Kerridge, J. F. Carbon, hydrogen and nitrogen in carbonaceous chondrites: Abundances and isotopic compositions in bulk samples. *Geochim. Cosmochim. Acta* **49**, 1707–1714 (1985).
5. Hashizume, K. & Sugiura, N. Nitrogen isotopes in bulk ordinary chondrites. *Geochim. Cosmochim. Acta* **59**, 4057–4069 (1995).
6. Petaev, M. I. The GRAINS thermodynamic and kinetic code for modeling nebular condensation. *Calphad* **33**, 317–327 (2009).
7. Lodders, K. Solar System Abundances and Condensation Temperatures of the Elements. *Astrophys. J.* **591**, 1220–1247 (2003).
